# Systematic review and network meta-analysis of performance of the Sapporo criteria, the revised Sapporo criteria, and the 2023 ACR/EULAR APS classification criteria for patients with antiphospholipid syndrome

**DOI:** 10.3389/fimmu.2026.1812710

**Published:** 2026-05-11

**Authors:** Yangqi Yin, Tianjiao Ma, Xinyue Zhang, Xuyang Chi, Kailin Zhu, Jizu Ling

**Affiliations:** 1 Department of Pediatrics, General Hospital of Fushun Mining Bureau of Liaoning Health Industry Group, Fushun, China; 2 Department of Pediatrics, The First Hospital of China Medical University, Shenyang, China; 3 Department of Hematology, Dalian Women and Children’s Medical Group, Dalian, China; 4 Second Department of Internal Medicine, School of Medicine, University of Occupational and Environmental Health, Kitakyushu, Japan; 5 Department of Pediatrics, The First Hospital of Lanzhou University, Lanzhou, China

**Keywords:** antiphospholipid syndrome, diagnostic criteria, diagnostic performance, network meta-analysis, systematic review

## Abstract

**Background:**

Antiphospholipid syndrome (APS) lacks systematic comparative evidence for the diagnostic performance of its three classification criteria, namely the 1999 Sapporo criteria, 2006 Revised criteria, and 2023 ACR criteria. This study aimed to comprehensively evaluate and compare the performance of the three criteria via a systematic review and network meta-analysis, providing an evidence-based basis for their clinical and research application.

**Methods:**

Following the PRISMA-NMA statement, we systematically searched PubMed, Embase, Cochrane Library, and Web of Science from inception to October 13, 2025, for studies evaluating the performance of the three APS classification criteria. Two reviewers independently performed study selection, data extraction, and quality assessment using the QUADAS-2 tool. Pairwise meta-analysis was conducted with Stata 15.0 to calculate the relative sensitivity and specificity. Network meta-analysis was performed using RStudio 4.3.0 to analyze sensitivity, specificity, DOR, and S index, and rank the diagnostic performance of the three criteria. Heterogeneity and publication bias were assessed using the I² index and Deeks’ funnel plot asymmetry test, respectively.

**Results:**

A total of 7 eligible studies involving 8 research cohorts (2,214 APS patients, 3,908 subjects) were included. In the direct pairwise meta-analysis of the 2006 Revised criteria versus the 2023 ACR criteria, the 2023 ACR criteria showed significantly lower sensitivity (relative sensitivity 0.80; 95% CI: 0.72–0.89; P < 0.01) and significantly higher specificity (relative specificity 1.06; 95% CI: 1.05–1.08; P < 0.01) compared with the 2006 Revised criteria. Network meta-analysis indicated that the 2006 Revised criteria had the highest sensitivity (0.86, 95% CI: 0.83-0.88) and S index (1.92, 95% CI: 0.33-3.00) among the three; the 2023 ACR criteria had the highest specificity (0.98, 95% CI: 0.97-0.98) and DOR (114.66, 95% CI: 75.46-168.19).

**Conclusions:**

The 1999 Sapporo criteria have limited clinical application value due to relatively poor diagnostic performance. The 2006 Revised Sapporo criteria have advantages in diagnostic sensitivity and comprehensive diagnostic performance (S index). The 2023 ACR/EULAR criteria exhibit superior specificity, making it well-suited for clinical research and as an adjunctive diagnostic tool for patients with non-classical APS manifestations.

**Systematic Review Registration:**

https://www.crd.york.ac.uk/prospero/, identifier CRD420251074199.

## 1 Introduction

Antiphospholipid syndrome (APS) is an autoimmune disease characterized by recurrent thrombotic events and adverse pregnancy outcomes in the presence of persistent positive antiphospholipid antibodies (aPLs). Due to the heterogeneity of clinical manifestations, standardized classification criteria are crucial for ensuring the homogeneity of patient groups in clinical research and improving the accuracy of clinical diagnosis. Since the first classification criteria for APS (1999 Sapporo) were proposed (1), two updated versions have been successively released: the 2006 Revised Sapporo criteria (2006 Revised) (2) and the 2023 ACR/EULAR APS classification criteria (2023 ACR) (3).

The 1999 Sapporo criteria require that for definite APS, at least one clinical criterion (vascular thrombosis or pregnancy morbidity) and one serologic criterion must be met; the relevant positive laboratory test must occur within five years of the clinical event; serologic criteria consist of lupus anticoagulant (LAC) positivity and/or medium-to-high titers of IgG/IgM anticardiolipin antibodies (aCL), with positivity confirmed on two occasions at least six weeks apart (1).

The 2006 revised criteria introduced significant adjustments to the 1999 Sapporo criteria: extending the interval between two positive test results from 6 weeks to 12 weeks, incorporating IgG/IgM anti-β2-glycoprotein I (aβ2GPI) antibodies into the laboratory criteria, and defining moderate-to-high titers of aCL or aβ2GPI as exceeding 40 GPL/MPL or the 99th percentile (2).

The 2023 ACR classification criteria for APS include six clinical domains and two serological domains, evaluated using a weighted scoring system. A formal APS classification requires a minimum of three points in each of the clinical and serological domains. Notably, these revised criteria have shortened the permissible interval between the clinical event and confirmatory laboratory testing from five years to three. The clinical criteria now incorporate a broader spectrum of manifestations, including established thrombotic risk factors, valvular heart disease, thrombocytopenia, and microvascular involvement. Furthermore, the definition of obstetric morbidity has been refined for greater specificity. Regarding aCL and aβ2GPI testing, the new criteria have shifted to using fixed thresholds (40–79 units for moderate positive; ≥80 units for high positive) and have discontinued the 99th percentile-based cut-off calculation (3).

The three classification criteria reflect a conceptual evolution in the definition of APS. The 1999 Sapporo and 2006 Revised criteria share a common conceptual framework centered on a binary “clinical + serological” paradigm, requiring at least one clinical criterion (vascular thrombosis or pregnancy morbidity) and one positive serological test. The 2006 revision expanded the serological panel and standardized positivity thresholds but retained the same fundamental structure. In contrast, the 2023 ACR criteria represent a paradigm shift, adopting a weighted, domain-based scoring system. Classification requires a minimum of 3 points in both the clinical and serological domains, with points accumulating across six clinical categories (including newly incorporated domains such as valvular heart disease, thrombocytopenia, and microvascular involvement) and two serological categories. Unlike the binary approach of earlier criteria, this cumulative model allows for a more detailed assessment and aims to better capture the heterogeneity of APS manifestations. A summary comparison of the three criteria is presented in **Table 1**.

**Table 1:** Conceptual comparison of the three APS classification criteria.

Feature	1999 Sapporo criteria	2006 Revised criteria	2023 ACR criteria
Classification requirement	≥1 clinical + ≥1 serological	≥1 clinical + ≥1 serological	≥3 points in clinical domain + ≥3 points in serological domain
Clinical domains	Vascular thrombosis, pregnancy morbidity	Vascular thrombosis, pregnancy morbidity	Six domains (includes venous thromboembolism, arterial thrombosis, microvascular involvement, cardiac valve involvement, pregnancy morbidity, thrombocytopenia and high-risk profiles for thrombosis)
Serological domains	LAC, aCL (medium-high titer)	LAC, aCL, aβ2GPI (medium-high titer, >99th percentile or >40 GPL/MPL)	LAC, aCL, aβ2GPI (weighted scoring based on fixed titer thresholds: 40–79 units for moderate, ≥80 units for high)
Time interval between clinical event and serological test	Within 5 years	Within 5 years	Within 3 years
Repeat serological test requirement	≥6 weeks apart	≥12 weeks apart	≥12 weeks apart (integrated into weighted scoring)

LAC, lupus anticoagulant; aCL, anticardiolipin antibodies; aβ2GPI, anti-β2-glycoprotein I.

Initially intended as a classification tool for APS, the 2006 revised criteria are now routinely applied for diagnosis in clinical settings. Despite the clear principle that classification and diagnostic criteria serve distinct purposes, this boundary is frequently overlooked in practice (4). Although the 2023 ACR criteria serve research objectives and may not be directly suitable for clinical diagnosis, they hold potential implications for clinicians’ diagnostic approaches. Therefore, a comparative analysis of the performance of the 1999 Sapporo criteria, the 2006 Revised criteria, and the 2023 ACR criteria is imperative, offering critical insights for clinical reference. Individual studies have shown that the 2023 ACR criteria have high specificity but relatively low sensitivity compared with the 2006 Revised criteria (5–7), but the results lack systematic verification. Network meta-analysis can integrate direct and indirect evidence from multiple studies to conduct comprehensive comparisons among multiple interventions (here, diagnostic criteria), providing more reliable evidence for clinical decision-making. Therefore, this systematic review and network meta-analysis aimed to comprehensively evaluate and compare the performance of the 1999 Sapporo criteria, 2006 Revised criteria, and 2023 ACR criteria, providing evidence-based basis for the selection of APS classification criteria in clinical and research practice.

## 2 Materials and methods

### 2.1 Protocol registration

This systematic review and network meta-analysis was conducted in accordance with the Preferred Reporting Items for a Systematic Review and Meta-analysis of Diagnostic Test Accuracy (PRISMA-NMA) statement (8). The study protocol was registered in the international prospective register of systematic reviews (PROSPERO) with the registration number CRD420251074199.

### 2.2 Eligibility criteria

Studies were included if they met the following criteria based on the Participants, Interventions, Comparisons, Outcomes (PICO) framework: (1) Participants: Patients with suspected or confirmed antiphospholipid syndrome (APS), including primary APS and secondary APS; (2) Interventions: Evaluation of the diagnostic performance of at least one of the three criteria (1999 Sapporo, 2006 Revised, 2023 ACR); (3) Comparisons: Direct or indirect comparisons of the performance among the three criteria; (4) Outcomes: the true positive (TP), false positive (FP), true negative (TN), and false negative (FN) cases.

Exclusion criteria: (1) Reviews, meta-analyses, case reports, letters, editorials, and animal studies; (2) Studies that could not obtain complete diagnostic performance data; (3) Non-English literature (due to language barriers in data extraction); (4) Studies with duplicate data or overlapping study populations; (5) The studies neither adhered to the standardized Enzyme-Linked Immunosorbent Assay (ELISA) protocol for aCL/anti-β2GPI or the International Society of Thrombosis and Hemostasis (ISTH)-recommended LAC testing (9), nor did they detail the methods for detecting aPLs.

### 2.3 Information sources and search strategy

A comprehensive electronic search was conducted in PubMed, Embase, Cochrane Library, and Web of Science databases from their inception to October 13, 2025. The search terms included combinations of “antiphospholipid syndrome”, “Sapporo”, “Sydney”, “revised”, “American College of Rheumatology”. The full search strategy is provided in Supplement **Table 2**. In addition, reference lists of included studies and relevant reviews were manually searched to identify additional eligible studies. No restrictions were imposed on the publication status (published or unpublished) to reduce publication bias.

**Table 2:** Direct pairwise meta-analysis of the 2023 ACR criteria versus the 2006 revised criteria.

Index test	Pooled sensitivity (95% CI)	Pooled specificity (95% CI)
Revised 2006	0.91 (0.84,0.95)	0.92 (0.90,0.93)
ACR 2023	0.73 (0.60,0.83)	0.98 (0.97,0.98)
The 2006 revised criteria versus the 2023 ACR criteria		
Relative Sensitivity (95% CI)	0.80 (0.72,0.89) *P value* < 0.01	
Relative Specificity (95% CI)	1.06 (1.05,1.08) *P value* < 0.01	

### 2.4 Study selection and data extraction

Two reviewers (YY and TM) independently screened the titles and abstracts of retrieved studies based on the eligibility criteria. For studies that met the preliminary screening criteria or had unclear information, full texts were retrieved for further evaluation. Disagreements between the two reviewers were resolved through discussion or consultation with a third senior reviewer (XZ). We used Endnote X9.1 software to record study selection decisions.

A pre-piloted data extraction form was used to extract data independently by the two reviewers, including: authors, publication year, country, sample size, and diagnostic data (TP, FP, TN, FN). If TP, TN, FP, or FN counts were unavailable in a study, we calculated them using the reported sensitivity, specificity, negative predictive value (NPV), or positive predictive valve (PPV).

### 2.5 Quality assessment

The quality of included diagnostic accuracy studies was evaluated using the Quality Assessment of Diagnostic Accuracy Studies-2 (QUADAS-2) tool (10), which includes four domains: patient selection, index test, reference standard, and flow and timing. Each domain was evaluated for the risk of bias, and the first three domains were also evaluated for concerns about applicability. Each item was rated as “low risk”, “high risk”, or “unclear risk”. The evaluation was conducted independently by two reviewers (YY and XZ), and disagreements were resolved through discussion. The risk of bias assessment was performed in Review Manager 5.3.

### 2.6 Statistical analysis

Conventional meta-analysis was performed using Stata 15.0 software with the “midas” package to calculate pooled sensitivity, specificity, positive likelihood ratio (PLR) and negative likelihood ratio (NLR). The summarize receiver operating characteristics (SROC) curves were constructed in Stata 15.0, and the area under the receiver operating characteristic curve (AUC) was calculated to reflect the overall diagnostic performance of each criterion. Pairwise meta-analysis was performed using Stata 15.0 software with the “metadta” package to calculate the pooled sensitivity and specificity of the 2023 ACR criteria and the 2006 Revised criteria, as well as the relative sensitivity and specificity of the 2006 Revised criteria versus the 2023 ACR criteria. We employed the random-effects ANOVA model proposed by Nyaga et al. for multiple comparisons of diagnostic tests (11). Network meta-analysis was performed using RStudio 4.3.0 with “Rstan” package. Markov Chain Monte Carlo (MCMC) sampling will be carried out with two chains, each consisting of 10,000 iterations, which includes a warm-up phase of 5,000 iterations and a thinning interval of 5. Convergence of the model was assessed with the potential scale reduction factor (R-hat). Good convergence was defined as an R-hat value below 1.1. Sensitivity, specificity, diagnostic odds ratio (DOR), and superiority index (S index) were used as the main outcome indicators. Using the superiority index, we ranked the performance of the three classification criteria. Heterogeneity among studies was evaluated using the Q statistic and I² index. I² > 50% or p < 0.10 indicated significant heterogeneity.

Publication bias was assessed using Deeks’ funnel plot asymmetry test; p > 0.05 indicated no significant publication bias.

## 3 Results

### 3.1 Study selection

**Figure 1:**
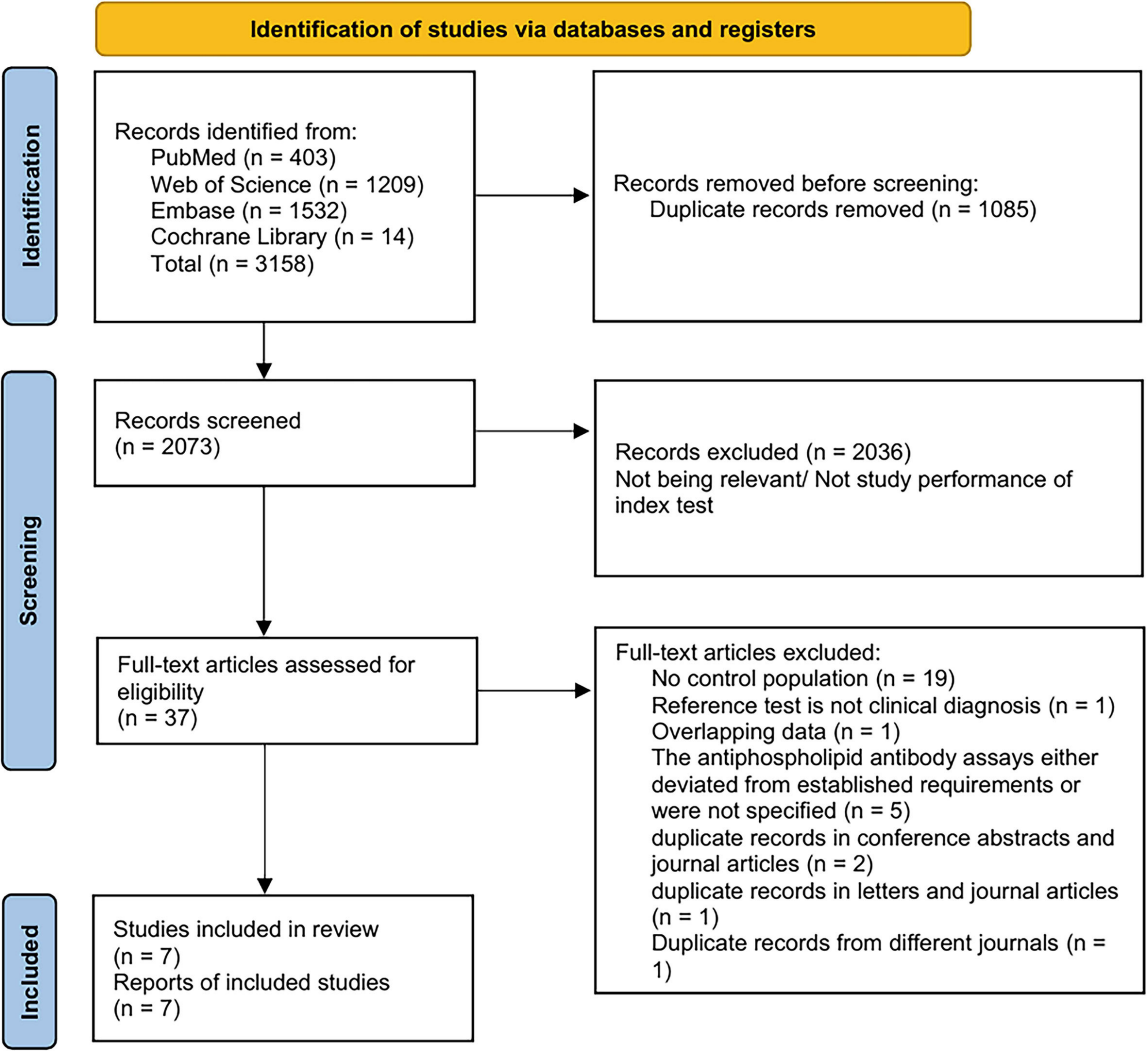
Flow chart of the study selection process.

A total of 3158 studies were retrieved through the initial electronic search. After removing 1085 duplicate studies, 2073 studies were screened for titles and abstracts, and 2036 studies were excluded. Thirty-seven studies were selected for full-text evaluation, and 30 studies were further excluded. Finally, 7 studies were included in the network meta-analysis (3, 6, 12–16), involving 8 research cohorts: Barbhaiya1/2023, Barbhaiya2/2023, Koliadenko/2024, Mısırcı/2024, Usta/2024, Yang/2024, Zhao/2024, and Estefanía/2025. The study selection process is shown in **Figure 1**.

### 3.2 Basic characteristics of included studies

The basic characteristics of the 7 included studies are summarized in **Supplementary Table 2**. All studies were published between 2023 and 2025, involving multiple regions (one from Europe and North America, one from Spain, two from China, one from Ukraine, and two from Turkey). The study designs included 2 cross-sectional studies and 5 cohort studies, with sample sizes ranging from 59 to 1443. A total of 3,908 subjects were included, comprising 2,214 patients with APS. All studies evaluated the diagnostic performance of the 2006 Revised criteria and 2023 ACR criteria, and 1 study also evaluated the 1999 Sapporo criteria.

### 3.3 Quality assessment results

**Figure 2:**
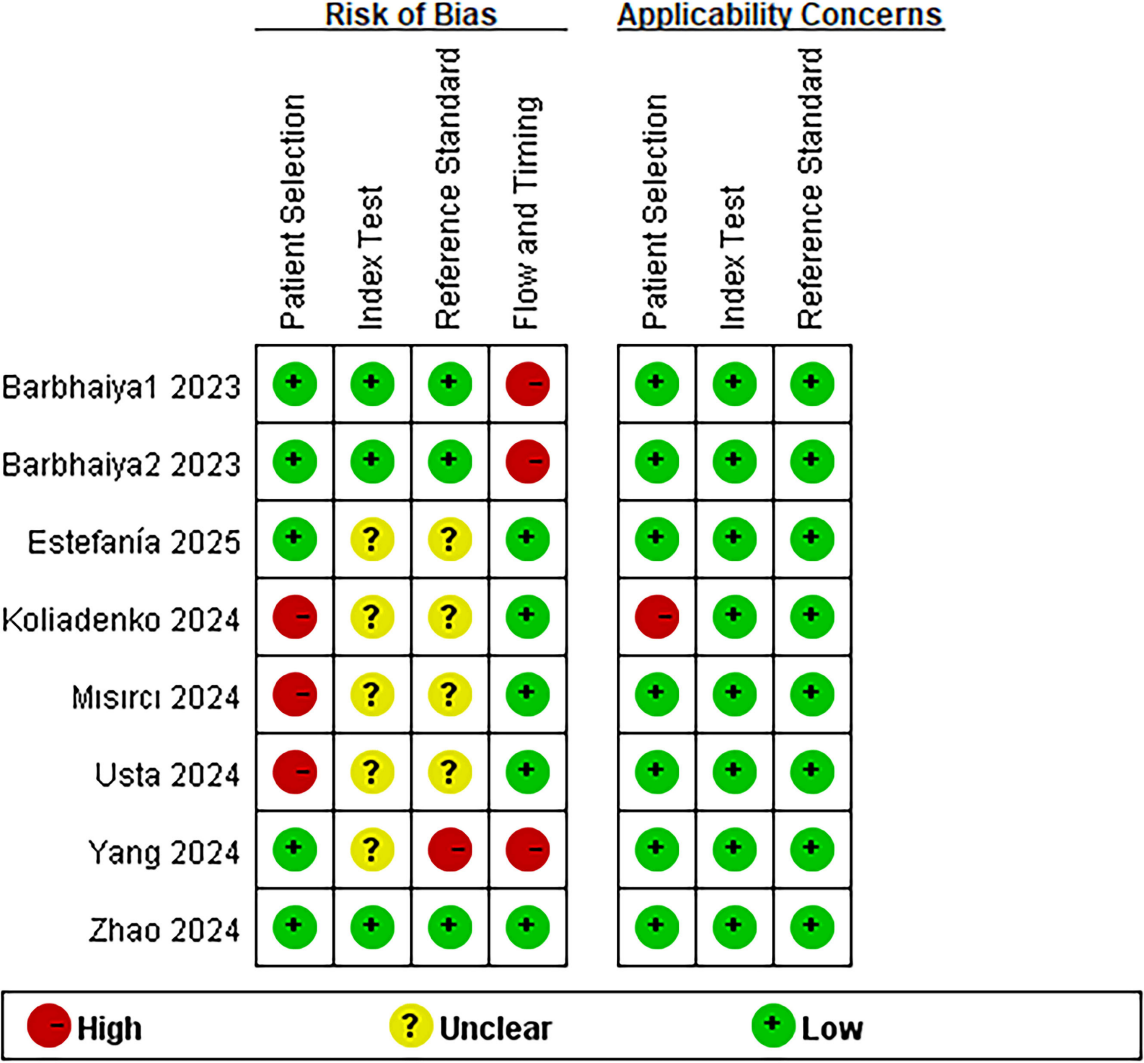
Quality assessment of diagnostic accuracy studies (QUADAS-2 tool) assessed the risk of bias and applicability concerns of the included studies.

The results of quality assessment using QUADAS-2 are shown in **Figure 2** and **Supplementary Figure 1**. In the patient selection domain, 3 studies were rated as having a high risk of bias due to their case-control design or inappropriate exclusion of cases. In the index test domain, 5 studies were rated as unclear risk of bias, as they did not specify whether blinding was applied during the interpretation of the index test findings. In the reference standard domain, 4 studies were rated as having an unclear risk of bias because they did not report whether blinding was maintained during interpretation of the reference standard results, while one study was judged as high risk because its reference standard interpretation was informed by the index test results. In the flow and timing domain, 3 studies were rated as high risk due to the exclusion of some enrolled cases from the analysis. With respect to applicability concerns, all studies demonstrated low concern in the three domains, with the exception of Koliadenko/2024, which was rated as high concern in the patient selection domain.

### 3.4 Conventional meta-analysis results

**Figure 3:**
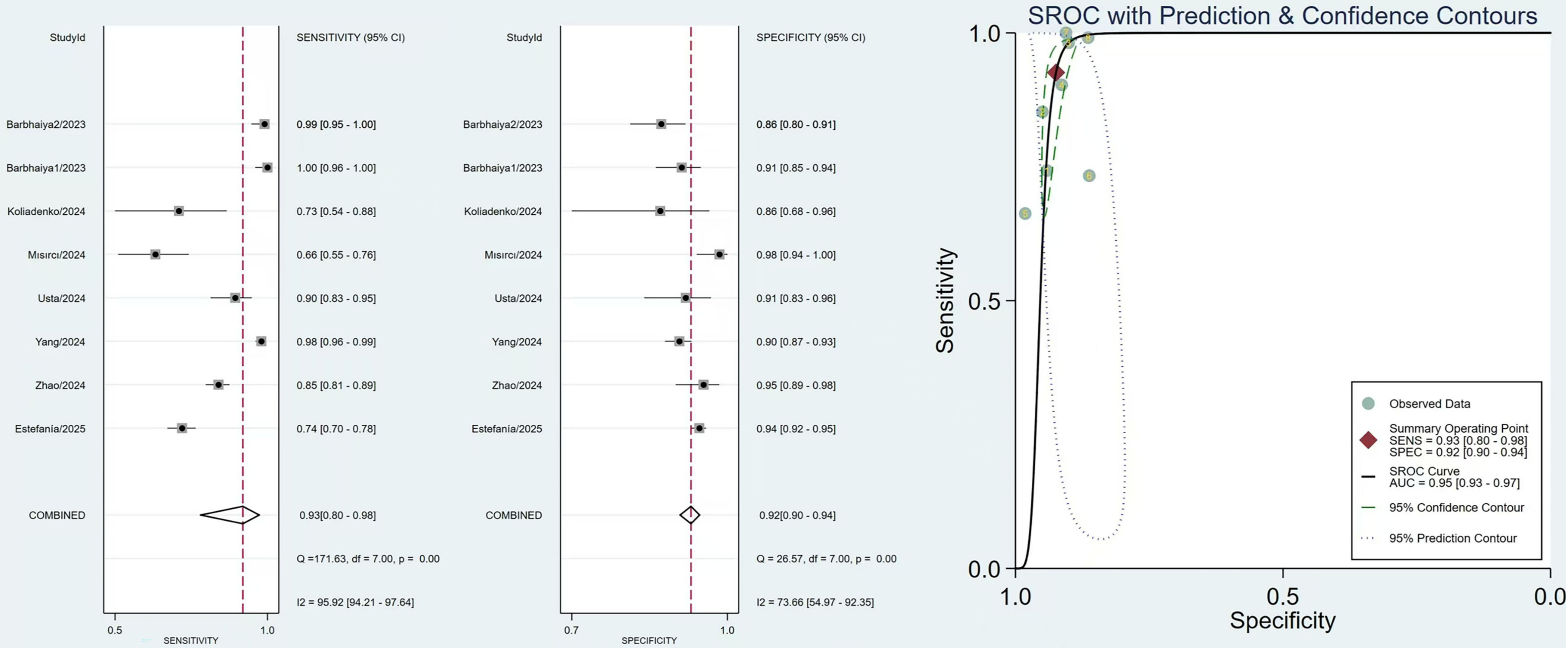
Paired forest plots of the sensitivity and specificity, and the SROC, summary receiver operating characteristic curve for the 2006 revised criteria.

**Figure 4:**
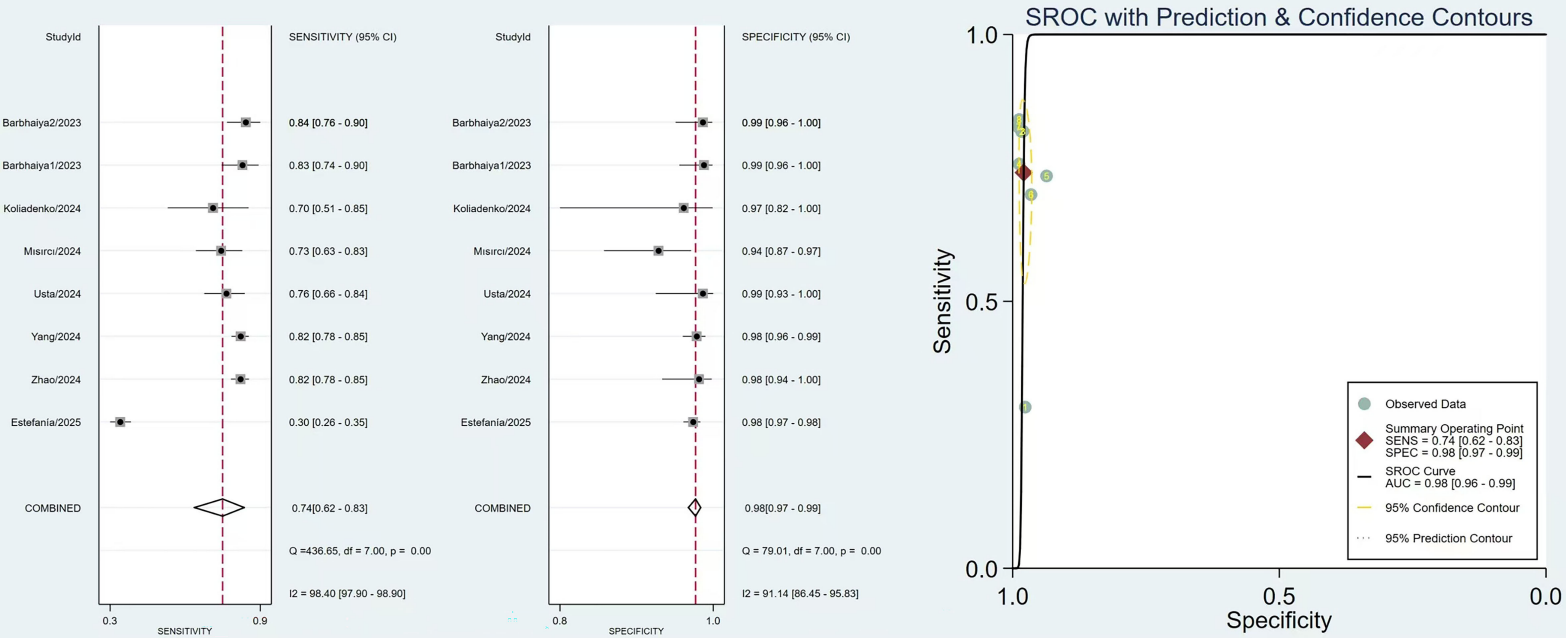
Paired forest plots of the sensitivity and specificity, and the SROC, summary receiver operating characteristic curve for the 2023 ACR criteria.

The Conventional Meta-Analysis results of sensitivity, specificity and SROC of the 2006 Revised criteria and the 2023 ACR criteria are shown in **Figures 3** and **4**. The pooled sensitivity of the 2006 Revised Sapporo criteria was 0.93 (95% CI: 0.80–0.98), and the pooled specificity was 0.92 (95% CI: 0.90–0.94). For the 2023 ACR criteria, the pooled sensitivity was 0.74 (95% CI: 0.62–0.83), and the pooled specificity was 0.98 (95% CI: 0.97–0.98). The AUC was 0.95 (95% CI: 0.93–0.97) for the 2006 Revised criteria and 0.98 (95% CI: 0.96–0.99) for the 2023 ACR criteria. The NLR, and PLR for the 2023 ACR criteria and the 2006 Revised criteria were shown in **Supplementary Figures 2**, **3**.

### 3.5 Direct pairwise meta-analysis

All seven included studies directly compared the diagnostic performance of the 2006 Revised criteria and the 2023 ACR criteria within the same patient populations, providing reliable paired data for the direct pairwise meta-analysis. The results of the direct pairwise meta-analysis comparing the 2006 Revised criteria and the 2023 ACR criteria are summarized in **Table 2** and **Supplementary Figure 6**. In the pairwise comparison, the relative sensitivity of the 2006 Revised criteria versus the 2023 ACR criteria was 0.80 (95% CI: 0.72–0.89; P < 0.01), indicating that the 2023 criteria had significantly lower sensitivity. The relative specificity was 1.06 (95% CI: 1.05–1.08; P < 0.01), demonstrating that the 2023 criteria achieved significantly higher specificity.

### 3.6 Exploratory network meta-analysis

Although the evidence network was limited—with all included studies directly comparing the 2006 Revised and 2023 ACR criteria, and only a single study evaluating the 1999 Sapporo criteria—we conducted an exploratory network meta-analysis to preliminarily compare the overall performance of all three criteria. The results of this exploratory analysis should be interpreted with caution, as the sparse evidence network limits the validity of indirect comparisons. The findings are summarized in **Table 3** and discussed below. The 2006 Revised criteria had the highest sensitivity (0.86, 95% CI: 0.83-0.88), which was higher than that of the 2023 ACR criteria (0.72, 95% CI: 0.69-0.75) and slightly higher than that of the 1999 Sapporo criteria (0.76, 95% CI: 0.39-0.96). In terms of specificity, the 2023 ACR criteria had the highest value (0.98, 95% CI: 0.97-0.98), which was significantly higher than that of the 2006 Revised Sapporo criteria (0.92, 95% CI: 0.90-0.93) and 1999 Sapporo criteria (0.89, 95% CI: 0.73-0.96). The S index of the 2006 Revised criteria (1.92, 95% CI: 0.33-3.00) was higher than that of the 2023 ACR criteria (1.68, 95% CI: 1.00-3.00) and the 1999 Sapporo criteria (0.75, 95% CI: 0.20-3.00). S index is a comprehensive indicator of diagnostic accuracy; a higher S index indicates better diagnostic performance. The DOR of the 2023 ACR criteria was the highest (114.66, 95% CI: 75.46-168.19), followed by the 2006 Revised criteria (70.06, 95% CI: 51.32-93.16) and the 1999 Sapporo criteria (59.20, 95% CI: 4.84-284.06).

**Table 3:** The Sapporo criteria, the revised criteria, and the 2023 ACR classification criteria for APS network meta-analysis results.

Index test	Sensitivity (95% CI)	Specificity (95% CI)	S index (95% CI)	DOR (95% CI)
1999 Sapporo	0.76 (0.39,0.96)	0.89 (0.73,0.96)	0.75 (0.20,3.00)	59.20 (4.84,284.06)
2006 Revised	0.86 (0.83,0.88)	0.92 (0.90,0.93)	1.92 (0.33,3.00)	70.06 (51.32,93.16)
2023 ACR	0.72 (0.69,0.75)	0.98 (0.97,0.98)	1.68 (1.00,3.00)	114.66 (75.46,168.19)

ACR, American College of Rheumatology; DOR, diagnostic odds ratio; S index, superiority index.

### 3.7 Heterogeneity analysis and publication bias

Considerable heterogeneity was observed among the included studies for both classification criteria. For the 2006 Revised criteria, the sensitivity demonstrated significant heterogeneity (Q = 171.63, I² = 95.92%, p < 0.01), as did the specificity (Q = 26.57, I² = 73.66%, p < 0.01). Similarly, for the 2023 ACR criteria, substantial heterogeneity was found in both sensitivity (Q = 436.65, I² = 98.40%, p < 0.01) and specificity (Q = 79.01, I² = 91.14%, p < 0.01). Deeks’ funnel plot asymmetry test was used to evaluate publication bias, and the results showed no significant publication bias for both the 2006 Revised criteria (p=0.75) and 2023 ACR criteria (p=0.31) (**Supplementary Figures 4**, **5**).

## 4 Discussion

This systematic review and network meta-analysis comprehensively compared the performance of the 1999 Sapporo criteria, 2006 Revised criteria, and 2023 ACR APS classification criteria, filling the research gap of insufficient direct comparative evidence for the three criteria in both clinical practice and research applications. The results clearly revealed the differential diagnostic characteristics of each criterion, providing an evidence-based basis for the rational selection of APS diagnostic tools in clinical settings and research.

The 2006 Revised criteria exhibited the highest diagnostic sensitivity among the three, which is consistent with the iterative optimization of this criterion on the basis of the 1999 version. The 2006 revision expanded the serological detection scope by incorporating aβ2GPI antibodies, and standardized the titer threshold of aPLs by defining moderate-to-high titers as exceeding the 99th percentile or 40 GPL/MPL (2). These adjustments made the criteria more inclusive of APS patients with diverse serological positive patterns, effectively reducing the rate of missed diagnoses caused by a single serological indicator. In addition, extending the interval between two positive serological tests from 6 weeks to 12 weeks improved the accuracy of identifying persistent aPLs positivity—the core serological feature of APS—while avoiding false negative results due to transient antibody positivity. The high sensitivity of the 2006 criteria makes it the optimal choice for preliminary screening of suspected APS populations (e.g., patients with unexplained thrombotic events, recurrent adverse pregnancy outcomes), as it can maximize the coverage of potential APS cases and lay a foundation for subsequent confirmatory diagnosis. The higher S index of the 2006 criteria further confirms its comprehensive advantage in balancing diagnostic identification ability, which is in line with its long-term status as the first-line clinical diagnostic criterion for APS worldwide.

The 2023 ACR criteria showed outstanding specificity and the highest DOR, reflecting its strong ability to distinguish APS from non-APS populations and reduce false positive diagnoses. This advantage stems from the fundamental innovation of the 2023 criteria in the classification framework: abandoning the traditional “clinical + serology” binary diagnostic model and adopting a weighted scoring system with six clinical domains and two serological domains, which requires a minimum of 3 points in both clinical and serological domains for classification. The scoring system refines clinical manifestations by incorporating vascular risk factors, valvular heart disease, thrombocytopenia, and microvascular involvement into clinical evaluation, and optimizes obstetric morbidity definitions to exclude non-APS-related adverse pregnancy outcomes, significantly improving the specificity of clinical phenotype identification. In terms of serology, the shift from a 99th percentile-based dynamic threshold to fixed titers (40–79 units for moderate positive, ≥80 units for high positive) for aCL and aβ2GPI antibodies reduces the variability of diagnostic thresholds caused by different laboratory reference ranges, and the weighted scoring of antibody titers (high positive with higher scores) further enhances the correlation between serological results and clinical APS characteristics. In addition, shortening the interval between clinical events and confirmatory serological testing from 5 years to 3 years makes the temporal correlation between clinical manifestations and serological positivity more stringent, reducing misdiagnosis caused by irrelevant aPLs positivity in the long term (3).

The finding that the 2023 criteria show lower sensitivity but higher specificity compared with the 2006 Revised criteria may appear counterintuitive. However, this pattern is consistent with the primary objective of the 2023 criteria: to serve as a rigorous classification tool for research, prioritizing high specificity to ensure homogeneous, well-characterized populations for high-quality epidemiological studies and clinical trials (3). Two key design features explain this trade-off. First, the weighted scoring system imposes a higher classification threshold, requiring at least 3 points in both clinical and serological domains. Patients with milder presentations—such as a single thrombotic event with moderate-titer aPL positivity—who would be classified under the 2006 criteria may not meet this stricter threshold. Second, the tightened temporal requirement (shortening the interval between clinical events and confirmatory serology from 5 to 3 years) enhances specificity by ensuring a close temporal link, though it may exclude some patients with remote but relevant events.

However, it is important to note that the implementation of fixed titer thresholds assume a level of harmonization across aPL detection methods that does not currently exist in clinical practice (17). There is no universally standardized ELISA or automated platform for aCL and aβ2GPI antibody testing, and fixed cutoffs (e.g., 40 units) may not correspond to equivalent biological positivity across different commercial assays or laboratory-developed tests (18–20). This lack of assay standardization likely contributes substantially to the heterogeneity observed across studies evaluating classification criteria, as variations in test performance—particularly in sensitivity and specificity—can directly affect classification outcomes. Future efforts should prioritize the harmonization of aPL detection methods to improve the consistency and applicability of these criteria across diverse clinical settings.

The 1999 Sapporo criteria, as the first standardized classification criteria for APS, laid the foundation for the diagnosis and research of this disease by establishing the core principle of “persistent aPLs positivity combined with clinical manifestations”. However, its diagnostic performance was the lowest among the three in this study, with relatively low sensitivity, specificity, S index and DOR, and a wide 95% confidence interval for most indicators. This is mainly due to the limitations of the 1999 version in serological detection and diagnostic thresholds: the only serological indicators included were LAC and aCL antibodies, missing a large number of APS patients with isolated aβ2GPI antibodies positivity; the short 6-week interval between two positive tests could not effectively exclude transient aPLs positivity induced by infection, medication and other factors, leading to both missed diagnoses and false positives. In addition, the small number of included studies evaluating the 1999 Sapporo criteria (only one study in this meta-analysis) may also lead to unstable result estimates, which further limits the clinical application value of this criterion in modern practice. At present, the 1999 criteria have basically been replaced by the 2006 revised version in clinical practice, and its main value is now reflected in historical research comparison and the construction of APS diagnostic criterion evolution system.

The significant heterogeneity observed in this study (high I² values for both sensitivity and specificity of the 2006 and 2023 criteria) is an important issue that needs to be noted. The sources of heterogeneity may be multi-faceted: first, the included studies cover diverse populations from Europe, North America, Spain, China, Ukraine and Turkey, and the differences in racial genetic background, clinical presentation spectrum of APS and laboratory detection standards may lead to inconsistent diagnostic results of the same criterion; second, there are differences in study design (cross-sectional studies and cohort studies) and sample size (59 to 1443 cases), which may affect the stability of diagnostic performance indicators; third, individual studies have different levels of bias in patient selection, index test interpretation and reference standard application (as shown in the QUADAS-2 quality assessment), such as case-control design bias, unblinded test interpretation, and exclusion of enrolled cases from analysis, which may interfere with the accuracy of data collection. Another important consideration pertains to the inherent differences in the conceptual frameworks underlying the 2006 Revised and 2023 ACR criteria, which may lead to the selection of distinct patient populations across validation studies. The 2023 ACR criteria incorporate a broader spectrum of clinical manifestations, including microvascular involvement, valvular heart disease, and thrombocytopenia—domains not explicitly captured by the 2006 Revised criteria (2, 3). Consequently, studies evaluating the performance of the 2023 ACR criteria are likely to enroll a higher proportion of patients presenting with these “non-classical” APS phenotypes. In contrast, studies assessing the 2006 Revised criteria predominantly include patients with classic thrombotic events or obstetric morbidity, as these constitute the core clinical requirements of the earlier framework. This fundamental difference in population composition represents a source of the substantial heterogeneity observed in our analyses.

Although subgroup analysis was not conducted due to the limited number of included studies, exploring the diagnostic performance of each criterion in specific subgroups (e.g., primary vs. secondary APS, thrombotic vs. obstetric APS, different age and gender groups) will be an important direction for future research to reduce heterogeneity and improve the applicability of results. Notably, this study confirmed no significant publication bias via Deeks’ funnel plot asymmetry test, which ensures the reliability of the meta-analysis results to a certain extent. The included studies were all published between 2023 and 2025, with high timeliness, and all of them adopted standardized aPLs detection methods (ELISA for aCL/aβ2GPI and ISTH-guided LAC testing), which reduced the bias caused by non-standard detection and improved the comparability of data among studies.

In addition, it is necessary to clarify the essential difference between classification criteria and diagnostic criteria. The three criteria evaluated in this study were initially formulated as classification criteria for homogenizing APS research populations, but the 2006 Revised Sapporo criteria have been widely used as diagnostic criteria in clinical practice due to their good sensitivity and operability. The 2023 ACR criteria, although currently positioned as a research classification tool, show great potential for clinical application due to its outstanding specificity. Clinicians should recognize this boundary and flexibly apply the criteria according to clinical needs: when conducting clinical research, the strict 2023 criteria should be used to ensure the homogeneity of the research population; when conducting clinical diagnosis, the 2023 ACR criteria are better suited as an adjunctive diagnostic tool for patients with atypical APS presentations, including those with isolated valvular heart disease, thrombocytopenia, or microvascular involvement, who might otherwise be missed by the 2006 criteria.

The three criteria evaluated in this study were originally developed to standardize patient selection for clinical research, ensuring homogeneity across study populations. However, in routine clinical practice, classification criteria are often used as de facto diagnostic tools, a pragmatic extension that is not without consequences. When classification criteria are applied for diagnostic purposes, their performance characteristics—particularly sensitivity and specificity—may be interpreted differently than intended by their developers. For instance, the high specificity observed for the 2023 ACR criteria likely reflects their deliberate design as a stringent classification tool aimed at minimizing false-positive inclusions in research settings. Conversely, the high sensitivity of the 2006 Revised criteria aligns with their widespread clinical use as a diagnostic benchmark, where the priority is to avoid missed cases. Readers should therefore interpret the reported performance metrics with this contextual nuance in mind: the figures represent how these criteria perform when used in a diagnostic capacity, which may differ from their performance when strictly applied as intended for research classification. Acknowledging this boundary is critical for appropriately translating study findings into both clinical practice and research protocol design.

This study has several limitations that need to be acknowledged. First, the total number of included studies is small (7 studies), and only one included study evaluated the 1999 Sapporo criteria, resulting in insufficient statistical power and wide 95% confidence intervals for its pooled estimates. Consequently, findings pertaining to the 1999 criteria should be interpreted with caution, and the core conclusions of this study are appropriately focused on the comparison between the 2006 Revised and 2023 ACR criteria, which represent the two standards most relevant to current clinical practice and research. Second, significant heterogeneity exists among the included studies, and the specific sources of heterogeneity cannot be fully explored and adjusted due to the limited number of studies, which may affect the generalizability of the results. Third, most of the included studies are observational studies, which may have potential selection bias and information bias, and lack the evidence from randomized controlled studies. Fourth, the included studies were limited to English literature, which may miss relevant research in other languages (e.g., Japanese, French, German) and lead to potential language bias. Fifth, this study did not evaluate the diagnostic performance of the three criteria in specific APS subgroups, nor did it analyze the impact of different aPLs detection platforms and laboratory quality control on diagnostic results, which needs to be supplemented by subsequent research. Sixth, the 2023 ACR criteria have a short application time, and the included studies are all short-term follow-up studies; the long-term diagnostic value and its impact on the prognosis of APS patients still need to be verified by long-term prospective studies. Additionally, the methodological approach of network meta-analysis warrants consideration. In this study, all included studies provided direct comparisons between the 2006 Revised and 2023 ACR criteria, whereas only a single study evaluated the 1999 Sapporo criteria. Consequently, the evidence network was extremely sparse, limiting the validity of indirect comparisons required for network meta-analysis. The network meta-analysis results should therefore be interpreted with caution.

## 5 Conclusions

In summary, the 1999 Sapporo criteria have limited clinical applicability owing to their relatively poor classification performance; the 2006 Revised criteria and the 2023 ACR criteria each offer distinct diagnostic advantages. The 2006 Revised criteria demonstrated the highest sensitivity and S index, whereas the 2023 ACR criteria exhibited superior specificity and the highest diagnostic odds ratio. The 2023 criteria are better suited for clinical research and may also serve as an adjunctive diagnostic tool for patients with non-classical APS manifestations.

## Data Availability

The original contributions presented in the study are included in the article/**Supplementary Material**. Further inquiries can be directed to the corresponding author.

## References

[1] Wilson WA Gharavi AE Koike T Lockshin MD Branch DW Piette JC . International consensus statement on preliminary classification criteria for definite antiphospholipid syndrome: report of an international workshop. Arthritis Rheumatism. (1999) 42:1309–11. doi: 10.1002/1529-0131(199907)42:7<1309::aid-anr1>3.0.co;2-f 10403256

[2] Miyakis S Lockshin MD Atsumi T Branch DW Brey RL Cervera R . International consensus statement on an update of the classification criteria for definite antiphospholipid syndrome (APS). J Thromb Haemostasis: JTH. (2006) 4:295–306. doi: 10.1111/j.1538-7836.2006.01753.x. PMID: 16420554

[3] Barbhaiya M Zuily S Hendry AM Manneville F Guillemin F Costenbader KH . 2023 ACR/EULAR antiphospholipid syndrome classification criteria. Ann Rheumatic Dis. (2024) 82:1258–70. doi: 10.1136/ard-2023-224609. PMID: 37989547

[4] Aggarwal R Ringold S Khanna D Neogi T Johnson SR Miller A . Distinctions between diagnostic and classification criteria? Arthritis Care Res. (2015) 67:891–7. doi: 10.1002/acr.22583. PMID: 25776731 PMC4482786

[5] Barbhaiya M Zuily S Naden R Hendry A Manneville F Amigo MC . The 2023 ACR/EULAR antiphospholipid syndrome classification criteria. Arthritis Rheumatol (Hoboken NJ). (2023) 75:1687–702. doi: 10.1136/ard-2023-224609. PMID: 37635643

[6] Yang Y Jiang H Tang Z Pan H Liu H Cheng X . Assessment of the 2023 ACR/EULAR antiphospholipid syndrome classification criteria in a Chinese cohort: Impact on clinical practice. J Autoimmun. (2024) 146:103237. doi: 10.1016/j.jaut.2024.103237. PMID: 38749076

[7] Lu Q Gan Y Yao Z Li C . A diagnostic performance study of the 2023 American College of Rheumatology/European Alliance of Associations for Rheumatology classification criteria for patients with antiphospholipid syndrome from the Antiphospholipid Syndrome Chinese Collaborative cohort presenting with suspected antiphospholipid syndrome. Arthritis Rheumatol (Hoboken NJ). (2024) 76:1317–8. doi: 10.1002/art.42835. PMID: 38403457

[8] McInnes MDF Moher D Thombs BD McGrath TA Bossuyt PM Clifford T . Preferred reporting items for a systematic review and meta-analysis of diagnostic test accuracy studies: the PRISMA-DTA statement. Jama. (2018) 319:388–96. doi: 10.1001/jama.2017.19163. PMID: 29362800

[9] Devreese KMJ De Groot PG De Laat B Erkan D Favaloro EJ Mackie I . Guidance from the Scientific and Standardization Committee for lupus anticoagulant/antiphospholipid antibodies of the International Society on Thrombosis and Haemostasis: Update of the guidelines for lupus anticoagulant detection and interpretation. J Thromb Haemostasis: JTH. (2020) 18:2828–39. doi: 10.1111/jth.15047. PMID: 33462974

[10] Whiting PF Rutjes AW Westwood ME Mallett S Deeks JJ Reitsma JB . QUADAS-2: a revised tool for the quality assessment of diagnostic accuracy studies. Ann Internal Med. (2011) 155:529–36. doi: 10.7326/0003-4819-155-8-201110180-00009. PMID: 22007046

[11] Nyaga VN Aerts M Arbyn M . ANOVA model for network meta-analysis of diagnostic test accuracy data. Stat Methods Med Res. (2018) 27:1766–84. doi: 10.1177/0962280216669182. PMID: 27655805

[12] Cantera Estefanía R Gálvez Sánchez R Abando Casuso M Flores García JA García Ruiz R Gorostidi Álvarez I . Assessment of 2023 ACR/EULAR antiphospholipid syndrome classification criteria in a Spanish cohort. Clin Chem Lab Med. (2025) 63:2272–81. doi: 10.1515/cclm-2025-0508. PMID: 40642778

[13] Zhao Y Huang C Zhou Y Qi W Cai B Hu C . Performance validation of the 2023 American College of Rheumatology/European League Against Rheumatism antiphospholipid syndrome classification criteria in an antiphospholipid syndrome cohort. J Thromb Haemostasis: JTH. (2024) 22:1660–74. doi: 10.1016/j.jtha.2024.02.019. PMID: 38462219

[14] Usta A Yayla ME Uslu E Sezer S Us E Ates A . The performance of 2023 American College of Rheumatology (ACR) / European Alliance of Associations for Rheumatology (EULAR) antiphospholipid syndrome classification criteria in a real-world rheumatology department. Mediterr J Hematol Infect Dis. (2024) 16:e2024074. doi: 10.4084/mjhid.2024.074. PMID: 39534708 PMC11556423

[15] Misirci S Ekin A Yagiz B Coskun BN Dalkilic E Pehlivan Y . The validation of the 2023 ACR/EULAR antiphospholipid syndrome classification criteria in a cohort from Turkey. Diagnostics. (2024) 14:2205. doi: 10.3390/diagnostics14192205 PMC11476238 39410609

[16] Koliadenko D Iaremenko O . Validation of 2023 ACR/EULAR antiphospholipid syndrome classification criteria in patients with systemic lupus erythematosus and positive antiphospholipid antibodies. Ann Rheumatic Dis. (2024) 83:542–3. doi: 10.1136/annrheumdis-2024-eular.4562

[17] Devreese KMJ Bertolaccini ML Branch DW De Laat B Erkan D Favaloro EJ . An update on laboratory detection and interpretation of antiphospholipid antibodies for diagnosis of antiphospholipid syndrome: guidance from the ISTH-SSC Subcommittee on Lupus Anticoagulant/Antiphospholipid Antibodies. J Thromb Haemostasis: JTH. (2025) 23:731–44. doi: 10.1016/j.jtha.2024.10.022. PMID: 39510414

[18] Vandevelde A Chayoua W De Laat B Gris JC Moore GW Musiał J . Semiquantitative interpretation of anticardiolipin and antiβ2glycoprotein I antibodies measured with various analytical platforms: Communication from the ISTH SSC Subcommittee on Lupus Anticoagulant/Antiphospholipid Antibodies. J Thromb Haemostasis: JTH. (2022) 20:508–24. doi: 10.1111/jth.15585. PMID: 34758192

[19] Vandevelde A Gris JC Moore GW Musiał J Zuily S Wahl D . Toward harmonized interpretation of anticardiolipin and anti-β2-glycoprotein I antibody detection for diagnosis of antiphospholipid syndrome using defined level intervals and likelihood ratios: communication from the ISTH SSC Subcommittee on Lupus Anticoagulant/Antiphospholipid Antibodies. J Thromb Haemostasis: JTH. (2024) 22:2345–62. doi: 10.1016/j.jtha.2024.04.016. PMID: 38704123

[20] Hu C Li S Xie Z You H Jiang H Shi Y . Comparison of different test systems for the detection of antiphospholipid antibodies in a Chinese cohort. Front Immunol. (2021) 12:648881. doi: 10.3389/fimmu.2021.648881. PMID: 34276646 PMC8283786

