# Supplementary material for: Systematic review and network meta-analysis of performance of the Sapporo criteria, the revised Sapporo criteria, and the 2023 ACR/EULAR APS classification criteria for patients with antiphospholipid syndrome
Source: Front Immunol. 2026 May 11;17:1812710. doi: 10.3389/fimmu.2026.1812710 (PMC13199258; doi:10.3389/fimmu.2026.1812710)
Supplement: Supplementary file 1 [file DataSheet1.pdf]

Content	Page
**Supplement Table 1**	2-3
**Supplement Table 2**	4-5
**Supplement Figure 1**	6
**Supplement Figure 2**	7
**Supplement Figure 3**	8
**Supplement Figure 4**	9
**Supplement Figure 5**	10
**Supplement Figure 6**	11

Supplement Table 1 Diagnostic characteristics of the included studies

Study/Year	Region	Index Text	TP	FP	FN	TN	APS cases	Control cases
Estefanía /2025	Spain	2023 ACR	128	24	296	995	424	1019
Estefanía /2025	Spain	2006 Revised	315	60	109	959	424	1019
Zhao/2024	China	2023 ACR	333	2	74	117	407	119
Zhao/2024	China	2006 Revised	347	6	60	113	407	119
Yang/2024	China	2023 ACR	357	10	79	504	436	514
Yang/2024	China	2006 Revised	428	51	8	463	436	514
Yang/2024	China	1999 Sapporo	397	48	39	466	436	514
Usta/2024	Turkey	2023 ACR	78	1	25	80	103	81
Usta/2024	Turkey	2006 Revised	93	7	10	74	103	81
Mısırcı/2024	Turkey	2023 ACR	61	7	22	103	83	110
Mısırcı/2024	Turkey	2006 Revised	55	2	28	108	83	110
Koliadenko/2024	Ukraine	2023 ACR	21	1	9	28	30	29
Koliadenko/2024	Ukraine	2006 Revised	22	4	8	25	30	29
Barbhaiya1/2023	Europe and	2023 ACR	81	2	17	178	98	180

	North America							
Barbhaiya1/2023	Europe and North America	2006 Revised	98	17	0	163	98	180
Barbhaiya2/2023	Europe and North America	2023 ACR	95	2	18	160	113	162
Barbhaiya2/2023	Europe and North America	2006 Revised	112	22	1	140	113	162

TP, true positive; FP, false positive; TN, true negative; FN, false negative; APS, Antiphospholipid syndrome

Supplement Table 2. Keywords applied in each database and results

	**Pubmed (October 12, 2025)**	
Search number	Query	Results
#1	Antiphospholipid Syndrome[Mesh]	9922
#2	Syndrome, Antiphospholipid[Title/Abstract] OR Anti-Phospholipid Antibody Syndrome[Title/Abstract] OR Antibody Syndrome, Anti-Phospholipid[Title/Abstract] OR Anti Phospholipid Antibody Syndrome[Title/Abstract] OR Syndrome, Anti-Phospholipid Antibody[Title/Abstract] OR Anti-Phospholipid Syndrome[Title/Abstract] OR Anti Phospholipid Syndrome[Title/Abstract] OR Syndrome, Anti-Phospholipid[Title/Abstract] OR Antiphospholipid Antibody Syndrome[Title/Abstract] OR Antibody Syndrome, Antiphospholipid[Title/Abstract] OR Antiphospholipid Antibody Syndromes[Title/Abstract] OR Syndrome, Antiphospholipid Antibody[Title/Abstract] OR Hughes Syndrome[Title/Abstract] OR Syndrome, Hughes[Title/Abstract]	2753
#3	#1 OR #2	11016
#4	American College of Rheumatology[Title/Abstract] OR ACR[Title/Abstract] OR European League Against Rheumatism[Title/Abstract] OR EULAR[Title/Abstract] OR Sapporo[Title/Abstract] OR Sydney[Title/Abstract] OR revised[Title/Abstract]	134564
#5	#3 AND #4	403
	**Web of Science (October 12, 2025)**	
#1	TS=(American College of Rheumatology OR ACR OR European League Against Rheumatism OR EULAR OR Sapporo OR Sydney OR revised)	370863
#2	TS=(Antiphospholipid Syndrome OR Anti-Phospholipid Antibody Syndrome OR Anti Phospholipid Antibody Syndrome OR Anti-Phospholipid Syndrome OR Anti Phospholipid Syndrome OR Antiphospholipid Antibody Syndrome OR Antiphospholipid Antibody Syndromes OR Hughes Syndrome)	24167
#3	#1 AND #2	1209
	**Embase (October 12, 2025)**	
#1	'antiphospholipid syndrome'/exp OR 'antiphospholipid syndrome'	25289

#2	'syndrome, antiphospholipid':ab,kw,ti OR 'anti-phospholipid antibody syndrome':ab,kw,ti OR 'antibody syndrome, anti-phospholipid':ab,kw,ti OR 'anti phospholipid antibody syndrome':ab,kw,ti OR 'syndrome, anti-phospholipid antibody':ab,kw,ti OR 'anti-phospholipid syndrome':ab,kw,ti OR 'anti phospholipid syndrome':ab,kw,ti OR 'syndrome, anti-phospholipid':ab,kw,ti OR 'antiphospholipid antibody syndrome':ab,kw,ti OR 'antibody syndrome, antiphospholipid':ab,kw,ti OR 'antiphospholipid antibody syndromes':ab,kw,ti OR 'syndrome, antiphospholipid antibody':ab,kw,ti OR 'hughes syndrome':ab,kw,ti OR 'syndrome, hughes':ab,kw,ti	4533
#3	#1 OR #2	26247
#4	'american college of rheumatology':ab,kw,ti OR 'acr':ab,kw,ti OR 'european league against rheumatism':ab,kw,ti OR 'eular':ab,kw,ti OR 'sapporo':ab,kw,ti OR 'sydney':ab,kw,ti OR 'revised'	232520
#5	#3 AND #4	1532
	**Cochrane Library (October 12, 2025)**	
#1	MeSH descriptor: [Antiphospholipid Syndrome] explode all trees	131
#2	(syndrome, antiphospholipid):ti,ab,kw OR (anti-phospholipid antibody syndrome):ti,ab,kw OR (antibody syndrome, anti-phospholipid):ti,ab,kw OR (anti phospholipid antibody syndrome):ti,ab,kw OR (syndrome, anti-phospholipid antibody):ti,ab,kw OR (anti-phospholipid syndrome):ti,ab,kw OR (anti phospholipid syndrome):ti,ab,kw OR (syndrome, anti-phospholipid):ti,ab,kw OR (antiphospholipid antibody syndrome):ti,ab,kw OR (antibody syndrome, antiphospholipid):ti,ab,kw OR (antiphospholipid antibody syndromes):ti,ab,kw OR (syndrome, antiphospholipid antibody):ti,ab,kw OR (hughes syndrome):ti,ab,kw OR (syndrome, hughes):ti,ab,kw	449
#3	#1 AND #2	14

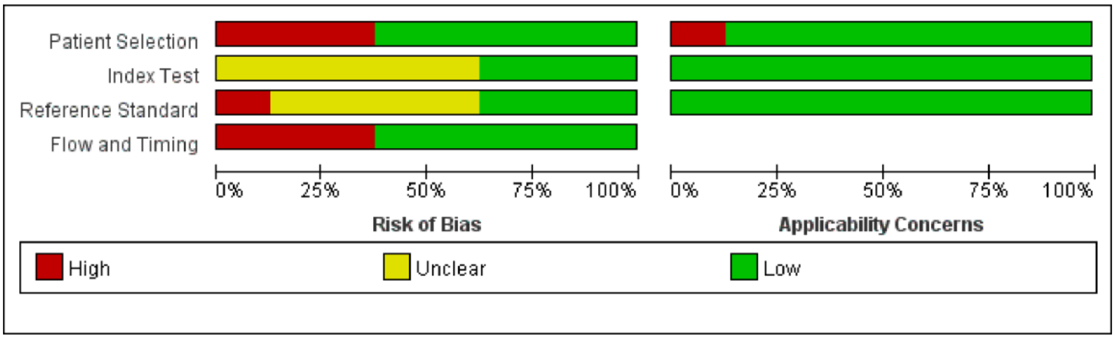

Supplement Figure 1 Quality Assessment of Diagnostic Accuracy Studies (QUADAS-2)

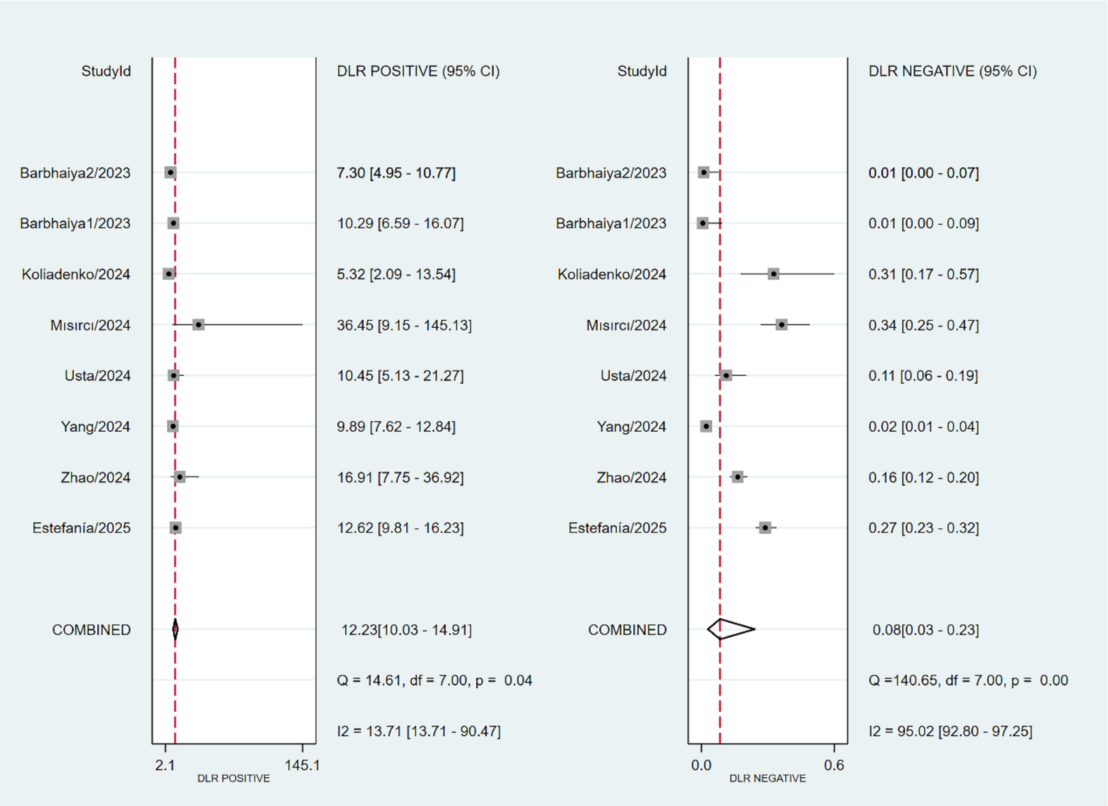

Supplement Figure 2. Paired forest plots of positive and negative likelihood ratios for the Revised criteria.

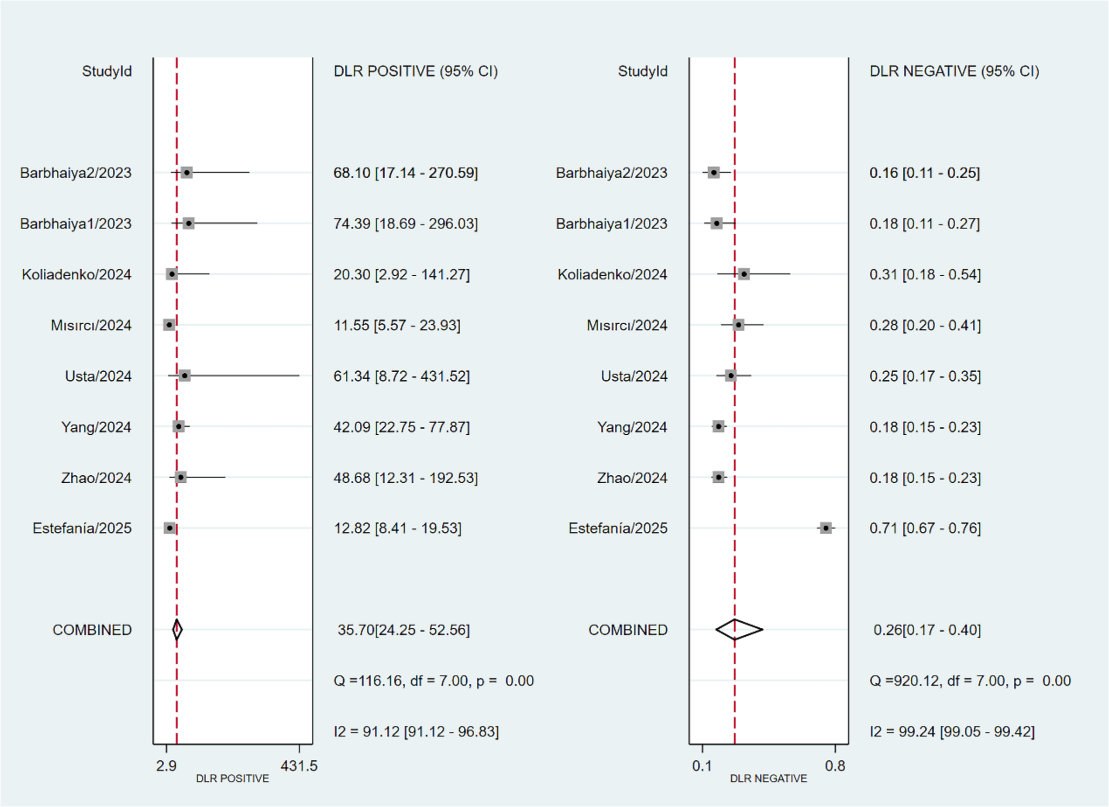

Supplement Figure 3. Paired forest plots of positive and negative likelihood ratios for the 2023 ACR classification criteria.

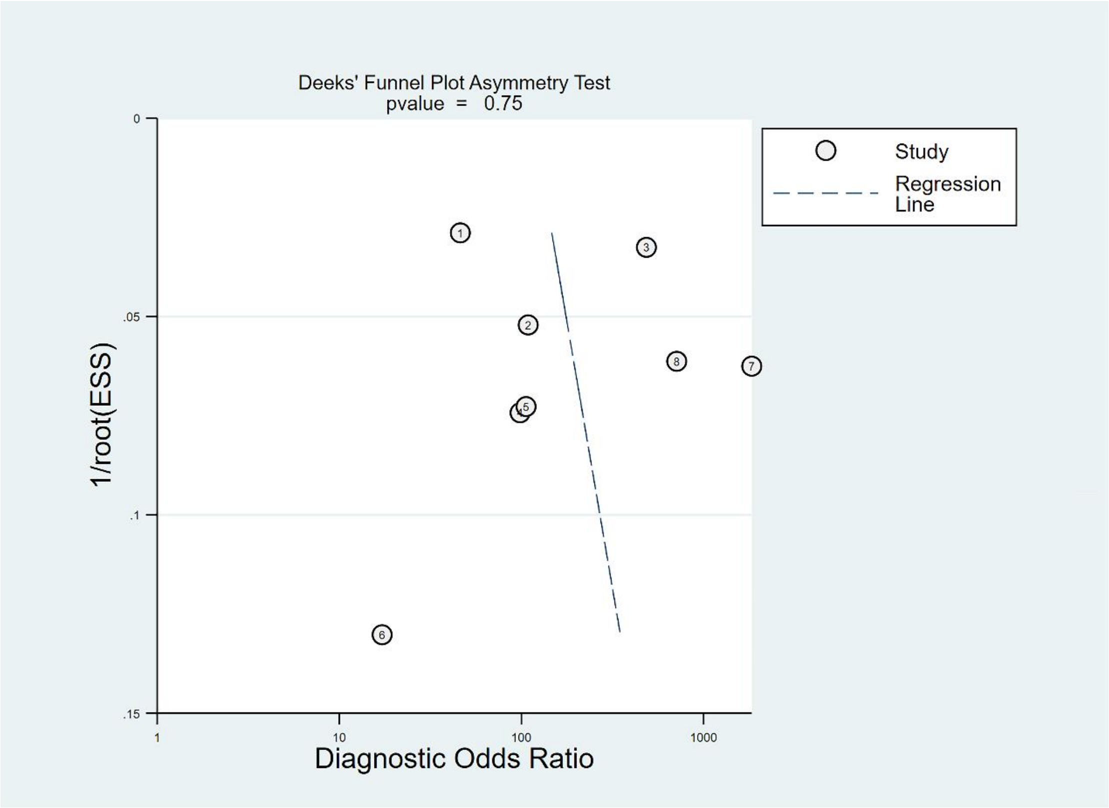

Supplement Figure 4. Deeks' funnel plot for the Revised classification criteria

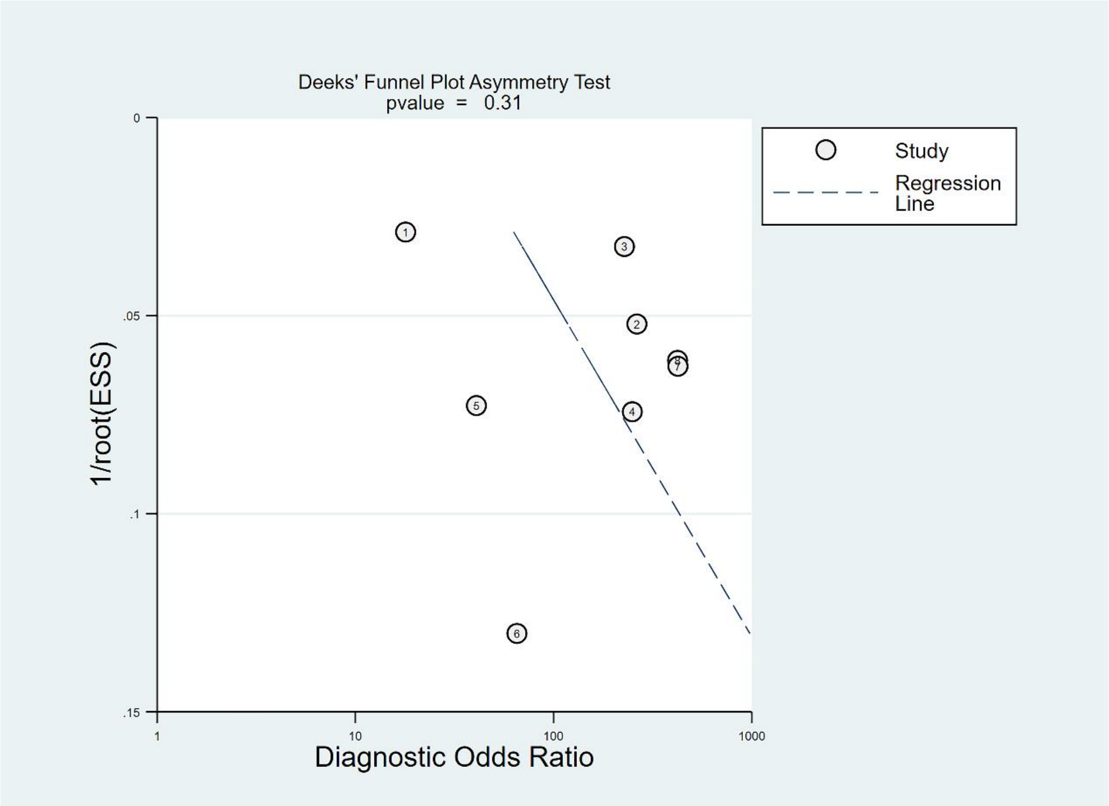

Supplement Figure 5. Deeks' funnel plot for the 2023 ACR classification criteria

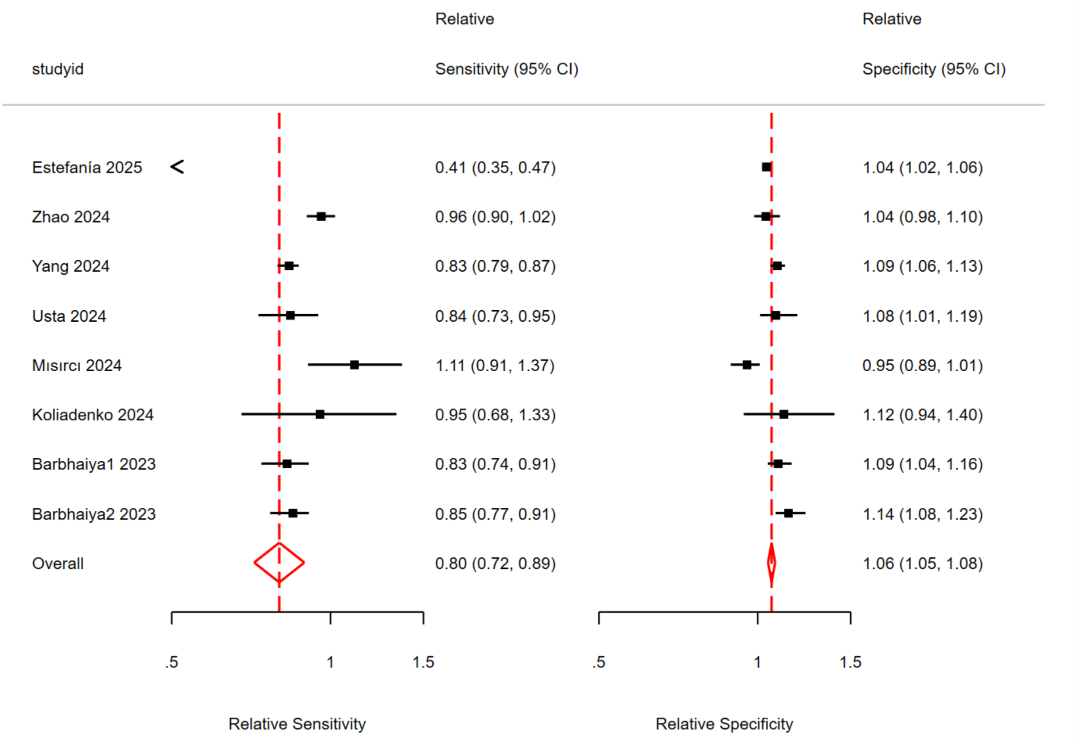

Supplement Figure 6. Forest plot of the relative sensitivity and relative specificity of the 2006 Revised criteria versus the 2023 ACR criteria.
